# Effects of maternal calcium and protein intake on the development and bone metabolism of offspring mice

**DOI:** 10.1515/biol-2022-0631

**Published:** 2023-07-06

**Authors:** Wenting Zhou, Tao Duan

**Affiliations:** Department of Obstetrics, Shanghai Key Laboratory of Maternal Fetal Medicine, Shanghai Institute of Maternal-Fetal Medicine and Gynecologic Oncology, Shanghai First Maternity and Infant Hospital, School of Medicine, Tongji University, No. 2699 West Gaoke Road, Shanghai 200092, China

**Keywords:** maternal diet, protein, calcium, osteoporosis, bone metabolism

## Abstract

Maternal nutrition is pivotal for offspring’s growth and development. Insufficient or unbalanced nutrition may cause osteoporosis and other diseases. Protein and calcium are essential dietary nutrients for offspring’s growth. However, the optimal contents of protein and calcium in maternal diet remain unclear. In the present study, we set four different protein and calcium content-pregnancy nutrition groups, including normal full-nutrient (Normal), low protein and low calcium (Pro−; Ca−), high protein and low calcium (Pro+; Ca−), and high protein and high calcium groups (Pro+; Ca+), to evaluate the weight gain of maternal mice as well as the weight, bone metabolism, and bone mineral density of offspring mice. When the vaginal plug is found, the female mouse will be kept in a single cage and fed with corresponding feed until delivery. The findings demonstrate that Pro−; Ca− diet affects the growth and development of offspring mice after birth. In addition, a low-calcium diet inhibits the growth of embryonic mice. Collectively, the present work further confirms the importance of protein and calcium in the maternal diet and deeply suggests their respective roles in different development stages.

## Graphical abstract

**Figure:**
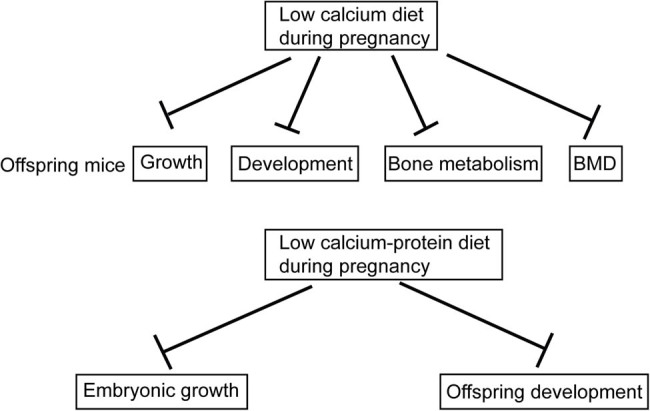

## 1 Introduction

For hundreds of years, the influences of maternal nutrition on fetal utero and postnatal growth have been the focus. In the 1990s, Prof. David Barker demonstrated that maternal nutritional deficiency increases the incidence of numerous diseases in offspring, including cardiovascular disease, diabetes, and other metabolic diseases [1]. Maternal intrauterine environments during pregnancy influence fetal programming during intrauterine growth, which not only impacts the growth and development but also generates permanent structural and functional changes [2,3,4], thus increasing the incidence of correlated diseases in the fetus when they grow up.

Osteoporosis is a systemic bone disease with a decrease in bone mass, bone mineral content, a destruction of bone microstructure, and an increase in bone fragility and fracture [5,6]; meanwhile, it can be affected by the environment [7]. With the aging of the world population, the incidence of osteoporosis is increasing [8,9]. Bone pain and fracture caused by osteoporosis decrease the quality of life in patients. Nutrition, especially protein and calcium, plays an essential role in maintaining bone mass [10,11]. As acknowledged, dietary risk factors of osteoporosis include insufficient intake of calcium, protein, and vitamin D [5,12,13,14].

The link/connection between osteoporosis in offspring and pregnant mice has been characterized, e.g., nutritional status during pregnancy influences offspring’s risk of osteoporosis in further life [15]. However, it remains unclear whether insufficient of protein and calcium during pregnancy will lead to abnormal development of newborns and children.

Herein, the nutritional osteoporosis animal model was utilized to study the effects of different calcium and protein intakes on the development and bone metabolism of offspring mice by limiting the nutritional intake of pregnant mice. The findings revealed the crucial roles of protein and calcium in pregnancy diet for the development and bone metabolism of offspring, thus further providing a reference for the establishment of optimal nutrition collocation during pregnancy.

## 2 Materials and methods

### 2.1 Animals

ICR mice were raised in the specific pathogen-free barrier system, with the room temperature of 23 ± 2°C, the relative humidity of about 60%, the lighting of 12 h light–dark cycle, and the ventilation rate of 20–50 times/h. The drinking water and bedding materials were sterilized by high-pressure steam, and freely available. The experimental feeds are configured and provided by Shanghai Shilin Biotechnology Co., Ltd. See Table 1 for the formula of relevant feed nutrients. Mice were randomly divided into four experimental groups according to different calcium and protein contents, i.e., normal full-nutrient group (normal, *n* = 20), low protein and low calcium group (Pro−; Ca−, *n* = 20), high protein and low calcium group (Pro+; Ca−, *n* = 20), and high protein and high calcium group (Pro+; Ca+, *n* = 20). During the experiment, each pregnant mouse was fed with a corresponding specific diet, while before pregnancy and after delivery, the mother mouse was fed with a normal diet.

**Table 1:** Feed nutrients

	Normal	Pro−; Ca−	Pro+; Ca−	Pro+; Ca+
Protein (%)	21	8	32	32
Calcium (%)	1.00	0.025	0.025	1.27


**Ethics approval:** The research related to animal use has been complied with all the relevant national regulations and institutional policies for the care and use of animals, and has been approved by the Ethics Committee of Shanghai First Maternity and Infant Hospital.

### 2.2 Breeding

Parental mice were 56 ± 2 days old and in normal health and mental state. Briefly, there were 10 males and 20 females in each group, with one male mouse and two female mice caged and fed with normal feed, the mating of mice was observed. When the vaginal plug was found, the female mouse would be kept in a single cage and fed with corresponding feed until delivery.

The pregnant mice per group are as listed: normal group (*n* = 17), Pro−; Ca− group (*n* = 18), Pro+; Ca− group (*n* = 18), and Pro+; Ca+ group (*n* = 19).

In total, 100 offspring mice per group were kept in the morning of the day after delivery to evaluate their body length.

### 2.3 Pregnancy observation

Pregnant mice were fed with a daily ration of 4 g corresponding feed and free drinking water according to different groups. Almost all of the feeds could be eaten up by mice; even though there were remaining feeds on the weighing plate, it was less than 0.2 g. The weight of pregnant mice was recorded once a week. On the day after delivery or in the morning of the next day, the birth weight and birth length of the pups were measured thrice (body length: the distance from the head to the anus of the mouse with left lying position, unit: mm); next, the average value was recorded.

### 2.4 Weight test

After delivery, mice in each group were fed with a normal diet. The birth weight of the new offspring mice was recorded in the morning of the next day after delivery and at a fixed time every week. To avoid nutrient deficiency caused by nutritional competition in offspring mice after birth, those cages with more than 8 offspring per litter were abandoned to 8 to continue breastfeeding. The young mice were fed to 3 weeks old and weaned. The offspring mice of each group were caged by gender. The mice per group are as listed: normal group (*n* = 80), Pro−; Ca− group (*n* = 80), Pro+; Ca− group (*n* = 80), and Pro+; Ca+ group (*n* = 80), with 40 female mice and 40 male mice in each group.

### 2.5 Fundus venous plexus blood collection

The offspring mice were fed to 8 weeks old and, thereafter, randomly selected for the collection of blood from the fundus venous plexus. The mice per group are as listed: normal group (*n* = 20), Pro−; Ca− group (*n* = 20), Pro+; Ca− group (*n* = 20), and Pro+; Ca+ group (*n* = 20), with 10 female mice and 10 male mice in each group. After being placed at 4°C for 1 h, the blood sample was centrifuged at 3,000 g for 15 min, followed by the collection of serum, which was stored at −20°C for further experimentation.

### 2.6 Serum calcium detection

The serum samples of mice were taken and sent to Shanghai Shenpu Pet Hospital. Serum calcium was measured with the full-automatic mouse serum calcium detector (IDEXX Laboratories, USA).

### 2.7 Serum pro-peptide of type I procollagen (PINP) detection

PINP biotin and diluted serum samples were mixed on a microplate mixer at 700 rpm for 1 h at room temperature. Then, the microplates were cleaned with sample diluent (SAMPDIL), followed by the addition of enzyme conjugate (Enzymconj), which was incubated at room temperature for 0.5 h. Afterwards, the microplates were cleaned with SAMPDIL, followed by the addition of TMB substrate, which was incubated at room temperature for 0.5 h. Thereafter, the reaction was stopped by HCl. Subsequently, the absorbance at 450 nm was recorded within 30 min. The standard curve was obtained by MultiCalc software (PerkinElmer). At last, PINP concentration was calculated by PINP detection kit (LDS, UK) based on the aforementioned absorbance and standard curve.

### 2.8 Serum TRACP 5b detection

Samples and release agents were added to the TRACP antibody-coated micropore, successively. After incubating and shaking at room temperature for 1 h, the substrate solution was added and incubated for 2 h at 37°C. Afterwards, NaOH solution was added to stop the reaction. Thereafter, the absorbance at 405 nm was read by MultiCalc software within 30 min. At last, the concentration of TRACP 5b was calculated by TRACP 5b detection kit (LDS, UK).

### 2.9 *In vivo* micro-CT detection

In brief, 1% pentobarbital sodium (80 mg/kg, Sigma Aldrich, USA) was injected intraperitoneally to induce anesthesia. Afterwards, mice were fixed on the GE Explore locus Micro-CT detection table with adhesive tape, to detect the bone mass, bone mineral density (BMD), and trabecular bone structure. The mice per group are as listed: normal group (*n* = 10), Pro−; Ca− group (*n* = 10), Pro+; Ca− group (*n* = 10), and Pro+; Ca+ group (*n* = 10), with 5 female mice and 5 male mice in each group.

### 2.10 Statistical analyses

Data were analyzed using GraphPad Prism 8.0 (USA) and stated as mean ± SD. Data were statistically compared by one‑way ANOVA followed by Tukey’s post hoc test. *p* < 0.05 was considered a significant difference.

## 3 Results

### 3.1 Low protein–calcium nutrient during pregnancy affects the weight of pregnant mice

**Figure 1:**
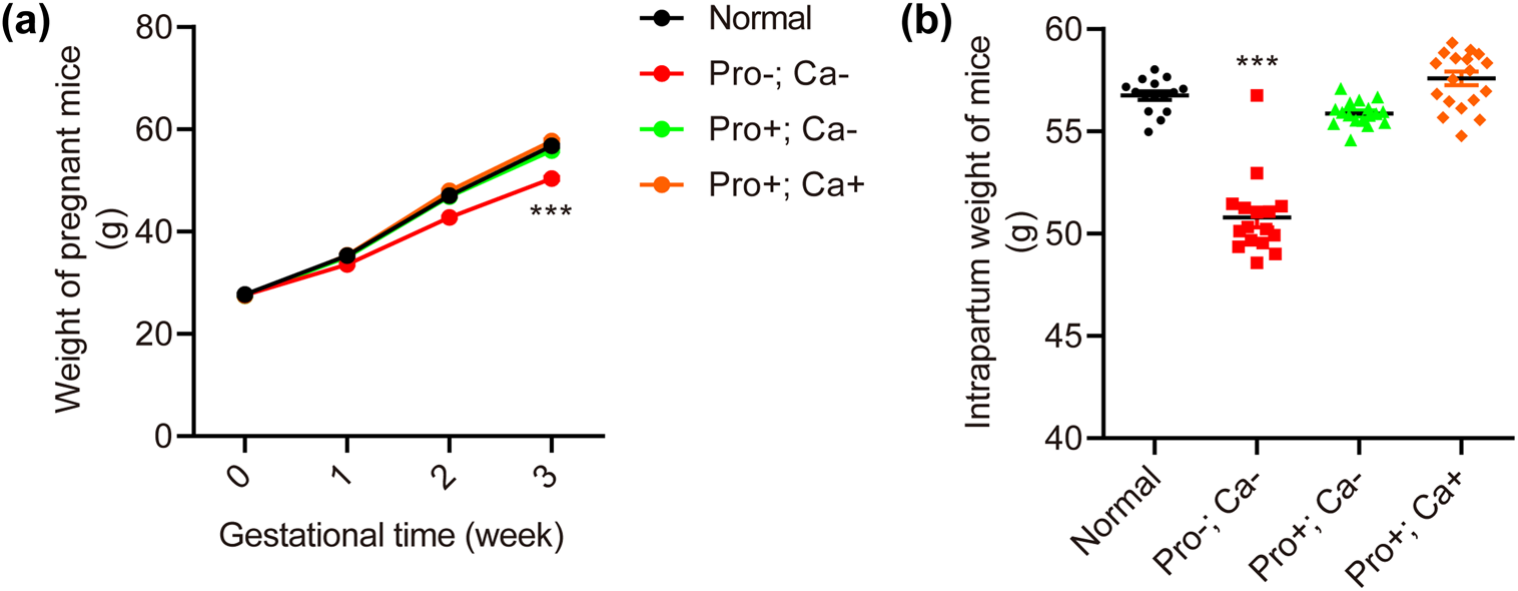
Low protein–calcium diet during pregnancy impacts the weight of pregnant mice. The weight of pregnant mice with Pro−; Ca− increases slowly compared with normal, Pro+; Ca−, and Pro+; Ca+ groups (a). The intrapartum body weight of pregnant mice with Pro−; Ca− (*n* = 18) is lighter than that of the normal group (*n* = 17) but not the Pro+; Ca− group (*n* = 18) or the Pro+; Ca+ group (*n* = 19) (b). ***p < 0.001.

The weight gain of pregnant mice during pregnancy was recorded (Figure 1a). As shown in Figure 1b, in the third trimester (3rd week of pregnancy), the weight of mice in the Pro−; Ca− group was significantly lower than that of the normal group, while no significant decrease was found between the Pro+; Ca− group and the normal group or the Pro+; Ca+ group and the normal group (normal: 56.75 ± 0.21 g; Pro−; Ca−: 50.79 ± 0.48 g, *p* < 0.001; Pro+; Ca−: 55.88 ± 0.14 g; Pro+; Ca+: 57.69 ± 0.33 g).

### 3.2 Low maternal protein–calcium nutrient impacts the development of newborn mice

**Figure 2:**
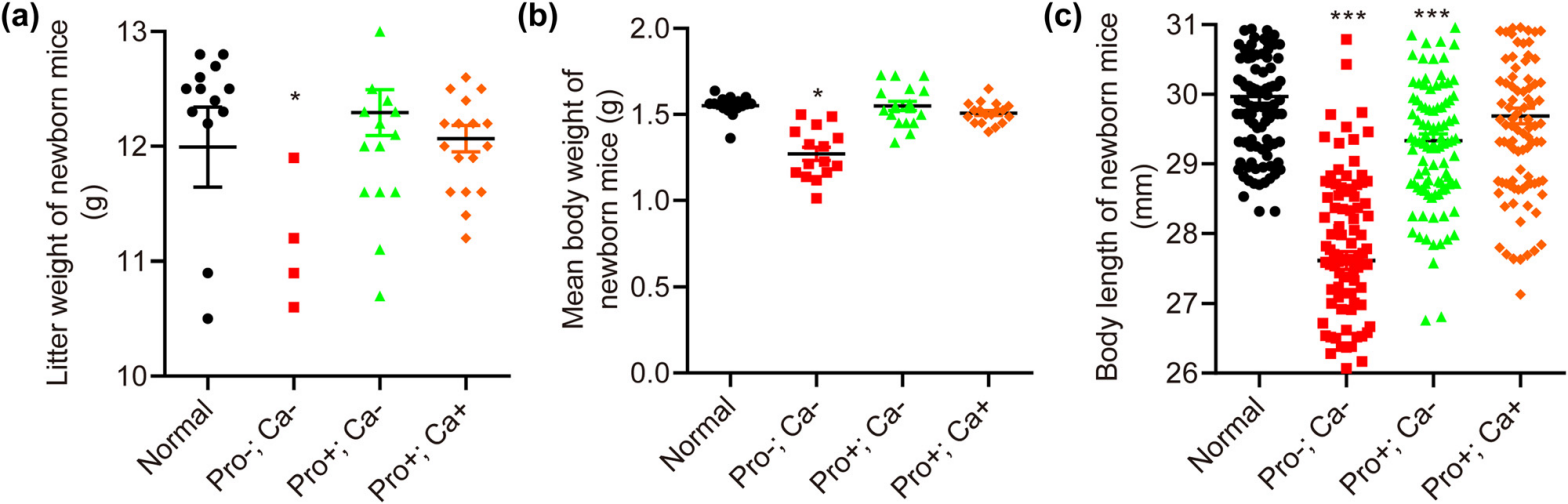
Low protein–calcium diet during pregnancy impacts the development of fetal mice. Except for the Pro+; Ca+ group, the litter weight (a), body weight (b), and body length (c, *n* = 100) of newborn pups in the Pro−; Ca− and Pro+; Ca− groups are significantly decreased compared to the normal group. *p < 0.05, ***p < 0.001. The number for (a) and (b) was the same as Figure 1, and listed as follows: normal group (*n* = 17), Pro−; Ca− (*n* = 18), Pro+; Ca− group (*n* = 18), and Pro+; Ca+ group (*n* = 19).

The litter weight of mice in each group was weighted (Figure 2a), which was listed as follows: normal: 11.99 ± 1.35 g; Pro−; Ca−: 9.11 ± 1.82 g, *p* < 0.05; Pro+; Ca−: 12.29 ± 0.85 g; and Pro+; Ca+: 12.07 ± 0.50 g. Among the four groups, the litter weight of newborn mice in the Pro−; Ca− group but not the Pro+; Ca− group or the Pro+; Ca+ group was significantly reduced compared with the normal group.

The mean birth weight of mice in each group was weighted (Figure 2b), which was listed as follows: normal: 1.55 ± 0.06 g; Pro−; Ca−: 1.27 ± 0.15 g, *p* < 0.05; Pro+; Ca−: 1.55 ± 0.11 g; Pro+; Ca+: 1.51 ± 0.06 g. Among the four groups, the birth weight of mice in the Pro−; Ca− group but not the Pro+; Ca− group or the Pro+; Ca+ group was significantly decreased compared to the normal group.

The birth length of mice in each group was also measured (Figure 2c), which was listed as follows: normal: 29.97 ± 0.10 mm; Pro−; Ca−: 27.61 ± 0.12 mm, *p* < 0.001; Pro+; Ca−: 29.33 ± 0.10 mm, *p* < 0.001; and Pro+; Ca+: 29.69 ± 0.11 mm. Among the four groups, the birth length of mice in both the Pro−; Ca− group and the Pro+; Ca− group but not the Pro+; Ca+ group was significantly shorter in contrast with the normal group.

### 3.3 Low maternal protein–calcium diet influences the development of offspring mice

**Figure 3:**
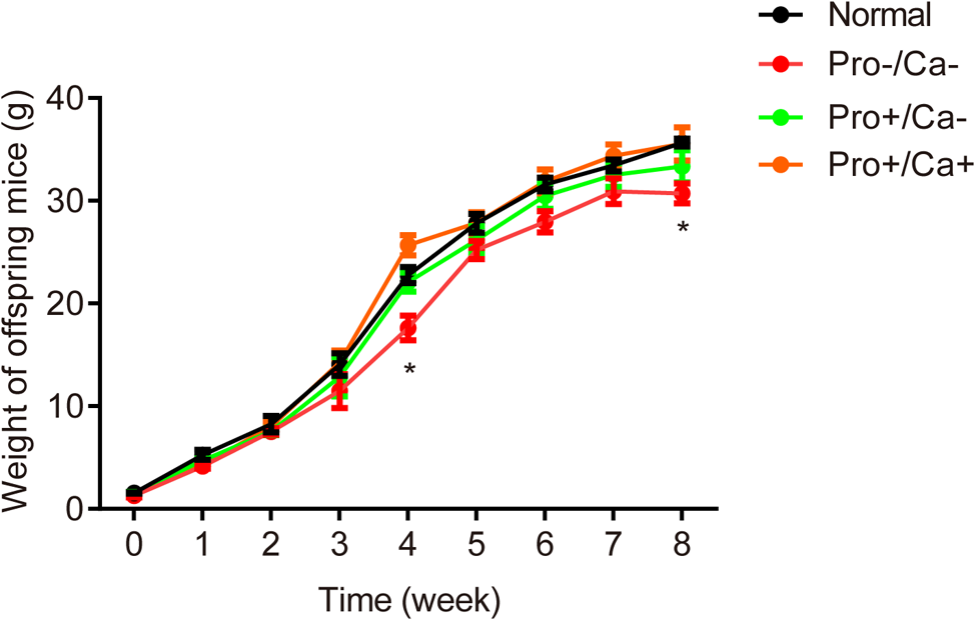
Low protein–calcium diet during pregnancy impacts the postnatal development of offspring mice. The body weights of offspring mice at weeks 3 and 8 were significantly lower in the Pro−; Ca− group in comparison with the normal group. *n* = 80. *p < 0.05.

As shown in Figure 3, at the age of 0–2 weeks, there was no significant difference in the weight of mice; when weaned at the age of 3 weeks, the weight of mice in the Pro−; Ca− group and the Pro+; Ca− group but not the Pro+; Ca+ group was significantly lower than that of the normal group (normal: 14.04 ± 1.10 g; Pro−; Ca−: 11.47 ± 1.64 g, *p* < 0.05; Pro+; Ca−: 12.86 ± 1.91 g, *p* < 0.05; Pro+; Ca+: 14.35 ± 1.17 g).

After weaning, mice in each group were fed with normal diet. Thereafter, the weight of the mice in the Pro−; Ca− and Pro+; Ca− groups increased slowly from week 4 to week 7, which were significantly different from the normal group at week 8, while there was no significant difference between the Pro+; Ca+ group and the normal group (normal: 36.10 ± 2.75 g; Pro−; Ca−: 30.67 ± 1.39 g, *p* < 0.05; Pro+; Ca−: 33.38 ± 0.24 g; and Pro+; Ca+: 35.52 ± 0.55 g).

### 3.4 Low maternal calcium diet affects the bone metabolism in offspring mice

**Figure 4:**
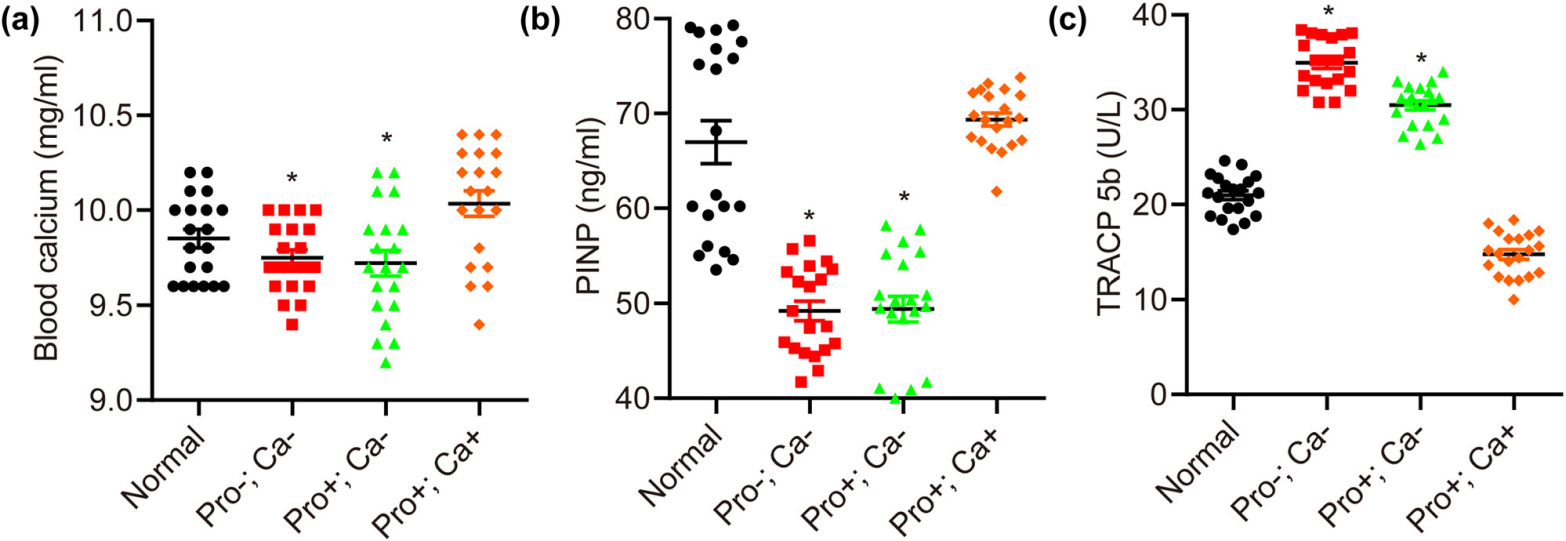
Low calcium diet during pregnancy interferes with the bone metabolism of offspring mice. The contents of blood calcium (a) and PINP (b) are significantly lower, while TRACP 5b (c) is significantly higher in offspring mice of the Pro−; Ca− and Pro+; Ca− groups compared with the normal group. *n* = 20. *p < 0.05.

As shown in Figure 4a, the serum calcium content in offspring mice of low calcium diet groups (Pro−; Ca− and Pro+; Ca−) but not Pro+; Ca+ group was significantly lower than that in the normal group (normal: 10.04 ± 0.10 mg/dl; Pro−; Ca−: 9.75 ± 0.05 mg/dl, *p* < 0.05; Pro+; Ca−: 9.72 ± 0.10 mg/dl, *p* < 0.05; Pro+; Ca+: 9.85 ± 0.07 mg/dl).

As presented in Figure 4b, there were significant decreases in serum PINP of the low calcium group (Pro−; Ca− and Pro+; Ca−) but not Pro+; Ca+ group, when compared with that in the normal group (normal: 66.99 ± 8.47 ng/ml; Pro−; Ca−: 49.21 ± 5.68 ng/ml, *p* < 0.05; Pro+; Ca−: 49.41 ± 8.16 ng/ml, *p* < 0.05; Pro+; Ca+: 69.41 ± 4.18 ng/ml).

As indicated in Figure 4c, serum TRACP 5b levels of mice in the low calcium diet groups (Pro−; Ca− and Pro+; Ca−) but not the Pro+; Ca+ group were significantly higher in comparison with the normal group (normal: 21.01 ± 2.07 U/l; Pro−; Ca−: 34.93 ± 2.44 U/l, *p* < 0.05; Pro+; Ca−: 29.99 ± 2.28 U/l, *p* < 0.05; Pro+; Ca+: 14.86 ± 2.45 U/l).

### 3.5 Low maternal calcium diet impacts BMD of offspring mice

**Figure 5:**
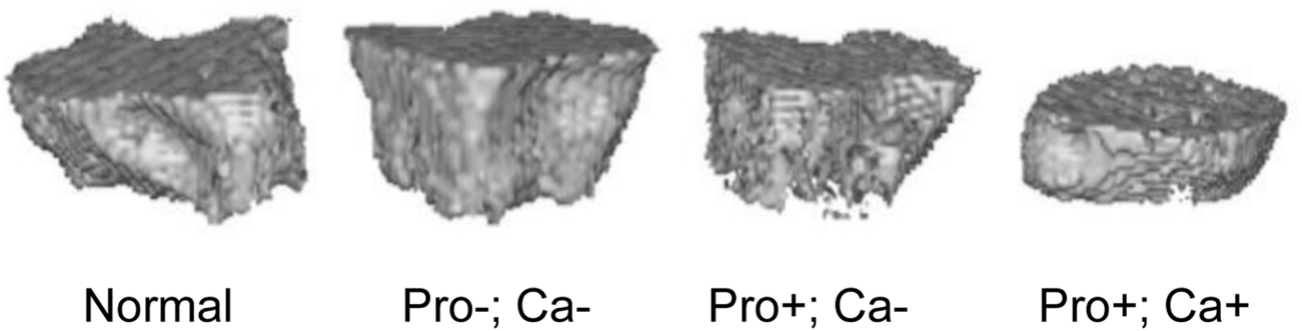
The left tibial metaphyseal growth plate in normal, Pro−; Ca−, Pro+; Ca−, and Pro+; Ca+ diet groups was observed by micro-CT. magnification, ×500. *n* = 10.

As shown in Figure 5, the left tibial metaphyseal growth plate was observed by micro-CT, with the gray level setting to 500, 2 layers below the growth plate as the starting point, and the next 16 layers for two- and three-dimensional (2D and 3D) image measurement. The volume of interest was drawn after getting the 2D image; thereafter, the corresponding BMD of cancellous bone and bone trabeculae was counted.

BMD and TMC of tibia metaphysis in low calcium diet groups (Pro−; Ca− and Pro+; Ca−) but not Pro+; Ca+ group were significantly lower than those in the normal group (Table 2).

**Table 2:** BMD of metaphysis of tibia ( \bar{x}\pm s] , *n* = 10)

Group	BMC (mg)	BMD (mg/cc)	TMC (mg)	TMD (mg/cc)	BVF (%)
Normal	0.54 ± 0.99	345.62 ± 0.56	0.53 ± 0.56	360.47 ± 0.78	0.93 ± 1.02
Pro−; Ca−	0.38 ± 0.74*	318.63 ± 1.08*	0.37 ± 0.86*	336.89 ± 1.02	0.91 ± 0.58
Pro+; Ca−	0.28 ± 1.07*	185.68 ± 1.05*	0.21 ± 1.03*	228.85 ± 0.64*	0.61 ± 0.96
Pro+; Ca+	0.49 ± 0.05	250.65 ± 1.23*	0.43 ± 0.58	298.05 ± 0.98*	0.73 ± 1.02

*p < 0.05.

Meanwhile, the trabecular bone microstructure parameters including Tb.Th., Tb.Sp., and SMI, in low calcium diet groups (Pro−; Ca− and Pro+; Ca−) but not in the Pro+; Ca+ group were significantly lower in comparison with the normal group (Table 3).

**Table 3:** Bone microstructure parameters of metaphysis of tibia ( \bar{x}\pm s] , *n* = 10)

Group	SMI	Tb.Th. (pixels)	Tb.Th. (mm)	Tb.Sp. (pixels)	Tb.Sp. (mm)
Normal	−45.69 ± 1.65	4.73 ± 1.02	0.21 ± 0.53	1.05 ± 0.84	0.05 ± 0.37
Pro−; Ca−	−16.84 ± 0.85*	2.15 ± 0.75*	0.09 ± 0.30*	1.07 ± 0.96	0.05 ± 0.43
Pro+; Ca−	−1.08 ± 1.02*	1.59 ± 0.68*	0.07 ± 0.32*	1.32 ± 0.67	0.06 ± 0.33
Pro+; Ca+	−4.00 ± 2.01*	2.85 ± 0.95	0.11 ± 0.40	1.32 ± 1.67	0.08 ± 0.71

*p < 0.05.

## 4 Discussion

As acknowledged, maternal nutrition status influences offspring’s risk of osteoporosis in further life [15]. Excessive or insufficient intake of certain nutrients during pregnancy may cause developmental disorders or malformations of fetal tissues and organs [16]. In the cell proliferation period, deficient maternal intake of calories or protein leads to stagnation of cell differentiation, insufficient number of cells in some organs of the fetus, and ultimately resulting in organ dysplasia or birth defects [17,18].

Calcium, an essential nutrient for bone construction, is an indispensable second messenger in signal transduction pathways, which regulate various processes, including gene expression, protein synthesis/secretion, muscle contraction, as well as body weight balance [19,20]. Placental transportation of calcium is pivotal for the development of fetal bone [21]. Protein, which makes up about 50% of bone volume and 33% of bone mass, provides the structural matrix of bone [22]. In light of novel researches and a plethora of available data on BMD, bone biomarkers, and fracture outcomes, the dietary protein intake was proved to be positively associated with bone health outcomes in healthy adults [23,24].

In the current study, the pregnant mice were divided into four groups by limiting the two nutrients of calcium and protein during the whole pregnancy period, demonstrating that when protein intake was low, not only the weight gain of the pregnant mice but also the birth weight of offspring mice were less than those of the normal group and protein-rich diet groups; however, when calcium intake was low, both low and high protein diet caused growth retardation and bone metabolism index reduction of offspring mice after weaning. In sum, the results demonstrate that adequate protein intake during pregnancy is crucial for the growth and development of fetal mice, while calcium is mainly involved in the bone metabolism and development of the offspring after birth, especially after weaning.

To explore the bone metabolism indicators and mechanism of maternal malnutrition affecting the postnatal development of mice, the contents of serum calcium [25,26], PINP [27,28], and TRACP 5b [29,30] were tested.

As acknowledged, low calcium intake declines the absolute absorption of calcium, a decrease in the source of calcium consumed by human body leads to a corresponding decrease of blood calcium; bone loss caused by a low calcium diet is mainly due to the enhancement of bone absorption [31]. Consistently, the serum calcium content in offspring mice of low calcium diet groups (Pro−; Ca− and Pro+; Ca−) but not Pro+; Ca+ group was lower than that in the normal group.

As well known, the formation of type I collagen, an important step in the process of bone formation, is the main organic component in the bone matrix. The amino terminal PINP is derived from type I procollagen during the process of collagen synthesis. Therefore, the quantitative determination of PINP reflects collagen synthesis and bone formation status [27,28]. In the current study, the PINP content in the low calcium diet groups (Pro−; Ca− and Pro+; Ca−) was lower than that of the normal group and the Pro+; Ca+ group, while no significant difference was observed between the Pro+; Ca+ group and the normal diet group. Altogether, a low calcium diet during pregnancy increased bone absorption and decreased bone formation rate in offspring mice, thus affecting bone development.

Serum TRACP 5b level was used to reflect the level of bone resorption [29,30], Herein, the TRACP 5b content in the offspring mice with low calcium, either low protein diet or high protein diet (Pro−; Ca− and Pro+; Ca−) was significantly upregulated, while in the offspring mice with Pro+; Ca+ diet was significantly downregulated, compared with the normal group. These results indicate that maternal calcium intake is negatively correlated with bone absorption and bone loss of the offspring mice after birth.

To confirm whether nutritional defects during pregnancy affect the BMD of offspring mice, micro-CT was used to detect the BMD and bone trabecula [32]. Also, micro-CT (Explore Locus) system was used for *in vivo* bone scanning in the present study, exhibiting that the BMD of the offspring mice with low calcium diets (Pro−; Ca− and Pro+; Ca−) was lower than that with a normal diet. SMI values of all three special feed groups (Pro−; Ca−, Pro+; Ca−, and Pro+; Ca+) were higher than those of the normal group, which indicated that the bone trabecular structure showed a “rod” development trend [33]. Trabecular thickness (Tb.Th.) of the three special feed groups (Pro−; Ca−, Pro+; Ca−, and Pro+; Ca+) was significantly lower than that of the normal group, while no significant difference in trabecular separation (Tb.Sp.) was observed among all the groups. These results demonstrate that low calcium and protein intake during pregnancy indeed affect the BMD and bone trabecular structure of offspring mice, while excessive protein and calcium intake during pregnancy may also exert negative effects on the bone trabecular structure of offspring mice.

In summary, a low calcium diet during pregnancy inhibits the growth, development, bone metabolism, and BMD of offspring mice after birth, while a low calcium–protein diet during pregnancy inhibits the embryonic growth and offspring development. Current research provides a reference for diet/nutrition matching during pregnancy and also supplies an experimental basis for researching the mechanism of osteoporosis in children.
